# Spheroids as a model for endometriotic lesions

**DOI:** 10.1172/jci.insight.160815

**Published:** 2023-06-08

**Authors:** Yong Song, Gregory W. Burns, Niraj R. Joshi, Ripla Arora, J. Julie Kim, Asgerally T. Fazleabas

**Affiliations:** 1 Department of Obstetrics, Gynecology and Reproductive Biology, Michigan State University, Grand Rapids, Michigan, USA.; 2 Department of Obstetrics and Gynecology, Feinberg School of Medicine, Northwestern University, Chicago, Illinois, USA.

**Keywords:** Reproductive Biology, Cell migration/adhesion

## Abstract

The development and progression of endometriotic lesions are poorly understood, but immune cell dysfunction and inflammation are closely associated with the pathophysiology of endometriosis. There is a need for 3D in vitro models to permit the study of interactions between cell types and the microenvironment. To address this, we developed endometriotic spheroids (ES) to explore the role of epithelial-stromal interactions and model peritoneal invasion associated with lesion development. Using a nonadherent microwell culture system, spheroids were generated with immortalized endometriotic epithelial cells (12Z) combined with endometriotic stromal (iEc-ESC) or uterine stromal (iHUF) cell lines. Transcriptomic analysis found 4,522 differentially expressed genes in ES compared with spheroids containing uterine stromal cells. The top increased gene sets were inflammation-related pathways, and an overlap with baboon endometriotic lesions was highly significant. Finally, to mimic invasion of endometrial tissue into the peritoneum, a model was developed with human peritoneal mesothelial cells in an extracellular matrix. Invasion was increased in the presence of estradiol or pro-inflammatory macrophages and suppressed by a progestin. Taken together, our results strongly support the concept that ES are an appropriate model for dissecting mechanisms that contribute to endometriotic lesion development.

## Graphical abstract

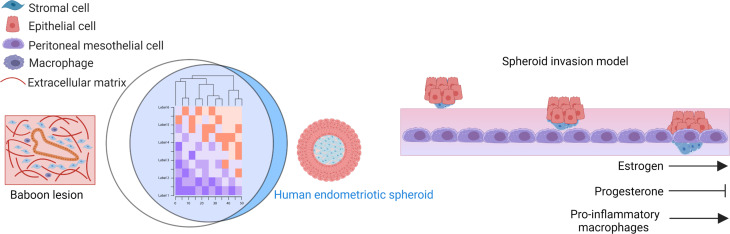

## Introduction

Endometriosis is a chronic, inflammatory, hormone-dependent disorder characterized by the presence of endometrial tissue outside the uterus (1). It affects between 10% and 15% of all women of reproductive age, 70% of women with chronic pelvic pain, and 20%–50% of women with infertility (2, 3). An average 7- to 10-year delay between the onset of symptoms and definitive diagnosis (4) places an enormous emotional and financial burden on both the patient and the health care system, with annual costs exceeding $78 billion each year in the United States alone (5, 6). Quality of life is reduced in women with endometriosis because of painful symptoms, which result in a loss of 11 hours of productivity per week (4). The etiology and pathogenesis of endometriosis remain unclear, but lesion development has been associated with cellular proliferation and invasion and interactions among endometriotic cells, immune cells, and their microenvironment (7, 8). Appropriate in vitro platforms that allow dissection of the underlying mechanisms of these processes will inform the understanding of the disease and further the development of novel therapeutics.

One of the major limitations to understanding the pathological events associated with endometriosis is the significant delay of 7–10 years for diagnosis and the subsequent variations in disease progression. Thus, both in vitro and appropriate in vivo models are indispensable to study the cellular and molecular mechanisms associated with endometriotic lesion development at different stages. The nonhuman primate and rodent models of endometriosis have been used most extensively for in vivo studies. While rodent models are advantageous because of the ability for tissue-specific gene manipulation and initial in vivo therapeutic testing (9), nonhuman primates offer a physiologically relevant model and provide an opportunity to study both the early events and disease progression (10, 11). Most in vitro studies use 2-dimensional monolayer cultures of primary or immortalized endometrial cells. This method allows for the study of endometriosis-associated mechanisms in a cell-specific manner and is easily manipulated for testing of treatments. However, the cell interactions are limited to the horizontal plane, and cells are exposed to a uniform concentration of factors. Therefore, monolayer cultures are unable to mimic the architecture and function of tissues in situ. Consequently, there is significant room for improvement of in vitro cell culture techniques to better mimic in vivo models. Three-dimensional (3D) cell culture systems, including organoids and spheroids, provide an exciting method to bridge the gap by incorporating more complex structures and multiple cell types.

Organoids are self-organizing, genetically stable, 3D culture systems containing both progenitor/stem and differentiated cells that resemble the tissue of origin (12–16). Spheroids are 3D cell aggregates, self-assembling in an environment that prevents attachment to a flat surface, that can mimic tissues and microtumors (17). Both organoids and spheroids are self-assembling 3D structures, while organoids are usually derived from primary or stem cells and are passageable. Boretto et al. established endometriotic epithelial organoids in a Matrigel culture system using ectopic lesions from patients with endometriosis and reported that the organoids replicated disease-associated functional and genomic traits (18). This system only allowed for the culture of endometriotic epithelial cells. However, the production of endometrial assembloids containing epithelium and stroma was possible when epithelial organoids were disrupted and recombined with stromal cells in a collagen hydrogel (19). Wiwatpanit et al. generated endometrial spheroids containing both epithelial and stromal cells from endometrial biopsies using micro-molded agarose plates, and the scaffold-free spheroids resembled the physiology of the normal endometrium (16). The micro-molded agarose plate spheroid culture system is more cost-effective and does not require exogenous scaffold materials but relies on de novo secretion of extracellular matrix and allows for self-organization and growth of multiple cell types. The potential for endometrial spheroids to serve as an improved in vitro model for endometriotic lesions is promising but has not been validated to our knowledge.

We aimed to develop a spheroid model of endometriotic lesions, containing both epithelial and stromal cell lines, that could be used as a platform to interrogate mechanisms of lesion invasion. Further, we posited that a combination of endometriotic epithelium and endometriotic stroma would align more closely with the transcriptome of spontaneous endometriotic lesions from the baboon than endometriotic epithelium with normal uterine fibroblasts. The baboon has been established as an appropriate model for human peritoneal endometriosis, supported by similar molecular changes in lesions and eutopic endometrium of women with endometriosis (20–26). The endometriotic epithelial cell line 12Z, simian virus 40–transformed endometriotic epithelial cells derived from peritoneal endometriosis, is widely used for the study of endometriosis (27) and was chosen for the epithelial component of spheroids. Immortalized human uterine fibroblasts (iHUFs) have been used extensively for in vitro decidualization experiments (26). We recently developed an endometriotic stromal cell line (iEc-ESC) from ovarian endometriosis (28). We hypothesized that the transcriptome of scaffold-free spheroids containing both endometriotic epithelial and stromal cell types would be similar to endometriotic lesions from the baboon model and support the use of endometriotic spheroids to explore peritoneal invasion associated with endometriotic lesion initiation.

## Results

### Epithelial and stromal cells self-organize in spheroids.

**Figure 1:**
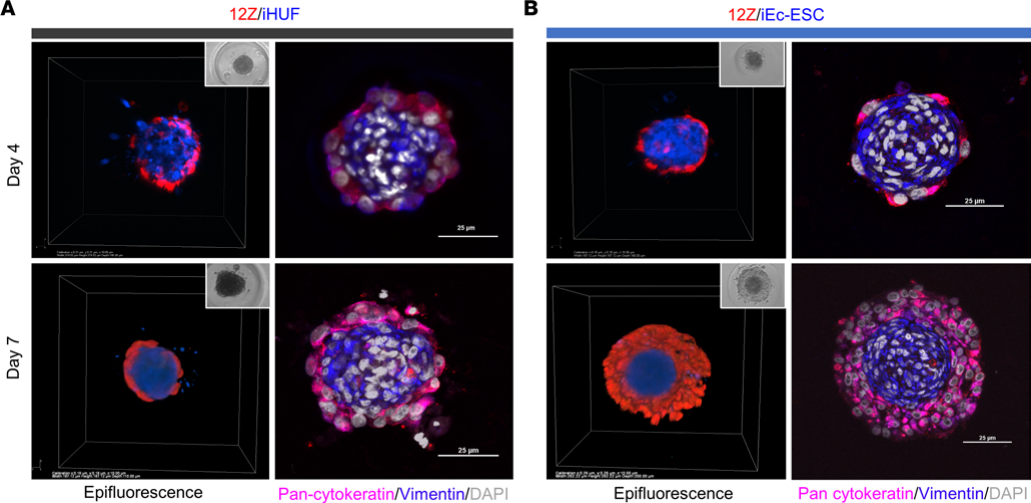
Spheroid structure. Spheroid development derived from (**A**) 12Z-RFP/iHUF-Azurite blue and (**B**) 12Z-RFP/iEc-ESC-Azurite blue (endometriotic spheroids). The left panels are 3D views and inserts are phase contrast images. The right panels are immunofluorescence staining for vimentin (shown in blue) and pan-cytokeratin (red). Scale bar: 25 μm.

Endometriotic lesions are hypothesized to develop from retrograde menstrual endometrial fragments, and epithelial and stromal cells are the 2 main cell types found in lesions. The cell lines employed in this study were immortalized ectopic epithelial cells (12Z), endometriotic stromal cells (iEc-ESCs), and normal human endometrial stromal cells (iHUFs) (26–28). For live visualization of cell type organization, epithelial 12Z cells were modified to express red fluorescent protein (RFP) and both stromal cell lines to express Azurite blue. Endometrial spheroids were generated from epithelial 12Z cells or stromal iEc-ESCs alone, 12Z+iHUFs, and 12Z+iEc-ESCs in micro-molded agarose well plates. A preliminary study empirically determined that the optimal ratio of epithelial cells to stromal cells for coculture was 1:50. Time-lapse imaging demonstrated that epithelial and stromal cells reorganized autonomously in the agarose wells (Supplemental Video 1; supplemental material available online with this article; https://doi.org/10.1172/jci.insight.160815DS1). The organization was verified by confocal image *Z*-stack reconstructions and immunohistochemical staining on days 4 and 7 (Figure 1). In 12Z/iHUF (Figure 1A) and 12Z/iEc-ESC (Figure 1B) spheroids, epithelial cells were located on the periphery of a stromal cell core by day 4. Endometriotic spheroids (ES) (12Z/iEc-ESC) developed multiple epithelial cell layers at day 7(Figure 1B), while 12Z/iHUF spheroids resulted in a single layer of epithelial cells (Figure 1A). Spheroids containing only epithelial 12Z cells or stromal iEc-ESCs formed simple structures (Supplemental Figure 1).

### Stromal cell origin alters spheroid gene expression.

**Figure 2:**
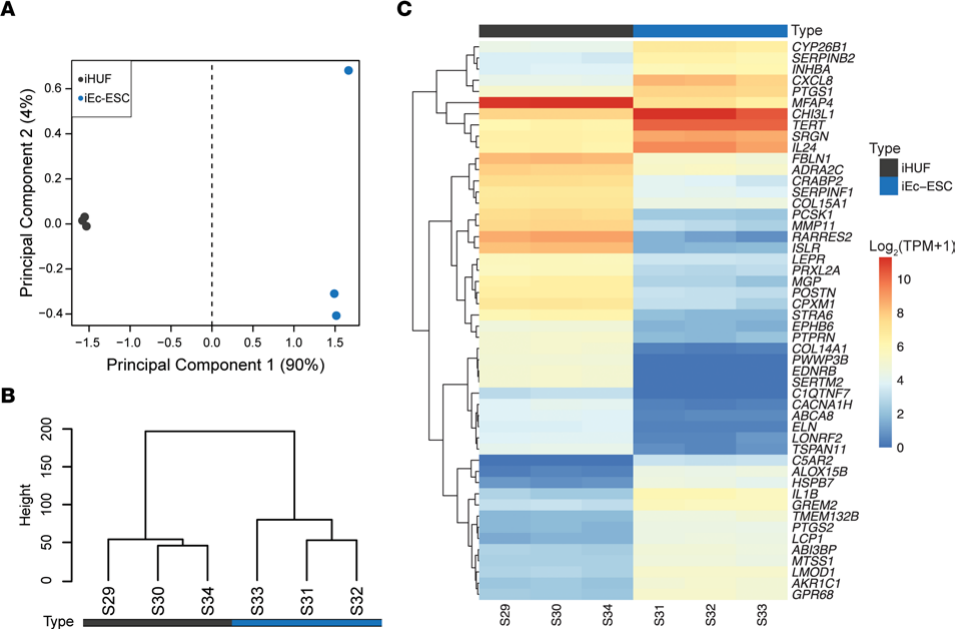
Spheroid gene expression is altered by stromal cell origin. (**A**) Principal component plot demonstrating transcriptome separation between 12Z/iEc-ESC (endometriotic spheroids, ES) and 12Z/iHUF spheroids. Legends indicate the difference in stromal cell origin: iHUF, uterine, or iEc-ESC, endometriotic. (**B**) Unsupervised hierarchical clustering dendrogram validating separation of sample groups and (**C**) a heatmap of the top 50 differentially expressed genes (edgeR-robust FDR *P* < 0.05) in ES with hierarchical gene clustering. *n* = 6. TPM, transcript per million.

Motivated by the phenotypic difference in spheroids with ESCs, we compared the transcriptome of ES, containing ectopic epithelial and stromal cell lines, with spheroids with normal stromal cells (12Z/iHUF). As an indication of broad transcriptome differences, ES and 12Z/iHUF spheroid samples were separated by principal component analysis across PC1 (65.7%, Figure 2A) and an unsupervised hierarchical clustering (Figure 2B). Indeed, there were 4,522 differentially expressed genes (DEGs), with 2,175 increased and 2,347 decreased, in ES compared with 12Z/iHUF spheroids. The top 50 DEGs included increased expression of interleukins (*CXCL8*, *IL1B*, *IL24*), prostaglandin synthase enzymes (*PTGS1*, *PTGS2*), and telomerase reverse transcriptase (*TERT*) (Figure 2C). Of note, stromal cell lines were immortalized by constitutive expression of *TERT* via lentiviral transduction, but *TERT* was not differentially expressed in an analysis of spheroids containing only stromal cells (Supplemental Figure 2), indicating the altered expression was due to an interaction between the epithelial and stromal cell types.

**Figure 3:**
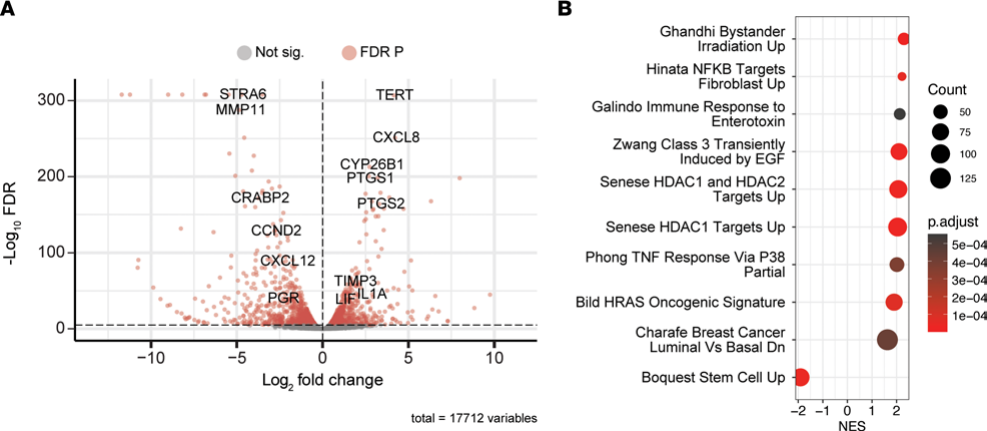
Transcriptome alterations in ES. (**A**) Volcano plot highlighting several of the top down- and upregulated genes (edgeR-robust FDR *P* < 0.05) in ES. (**B**) The top MSigDB C2 enriched gene sets (GSEA FDR *P* < 0.05) from ES. A positive normalized enrichment score (NES) indicates activation, or increased expression, of the gene set. *n* = 6.

Prostaglandin synthase enzymes *PTGS1* and *PTGS2*; *CXCL8*; *TIMP3*, an inactivator of extracellular matrix remodeling enzymes; *IL1A*; and leukemia inhibitory factor (*LIF*), an estrogen response gene, were increased in ES while *STRA6* and *CRABP2*, retinoic acid–binding proteins, and *PGR* were downregulated (Figure 3A and Supplemental Table 1). Altered gene expression was further characterized by gene set enrichment analysis (GSEA) using the Molecular Signatures Database (MSigDB) C2 data sets. These gene sets are curated from multiple sources, including published research describing the outcome of chemical and genetic perturbations and canonical pathways. The results included NF-κB target genes, 2 senescence gene sets, and TNF-α response via p38 mitogen-activated protein kinases as the most highly increased gene sets for ES, while stem cell–associated genes, including *CDKN2C*, *CDH11*, and *ROR1*, were decreased (Figure 3B). The activation of NF-κB signaling was concentrated in downstream genes associated with the canonical pathway promoting survival and inflammation based on the Kyoto Encyclopedia of Genes and Genomes (KEGG) NF-KAPPA B signaling pathway (Supplemental Figure 3).

### ES are similar to lesions.

**Figure 4:**
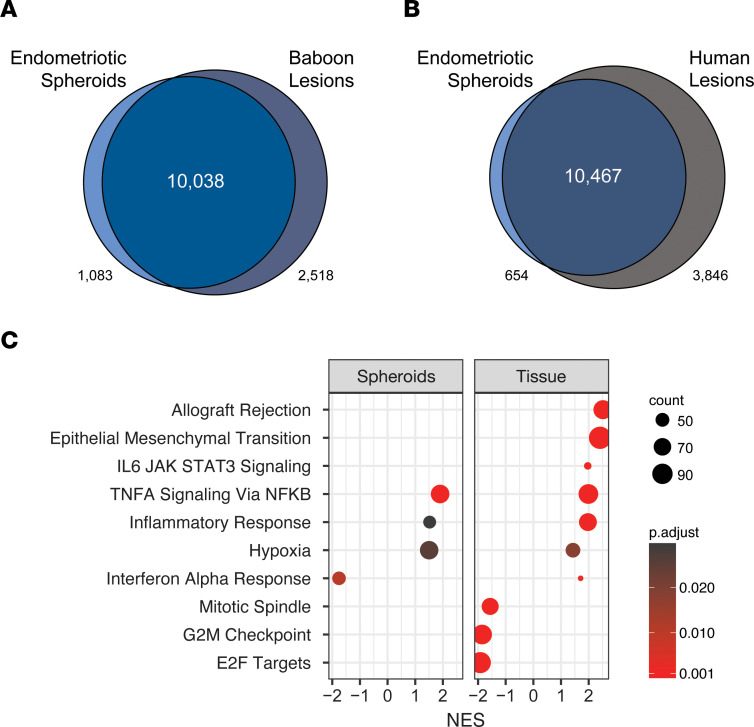
ES and lesion gene expression are similar. (**A**) Overlap of genes expressed in ES (*n* = 3) with baboon lesions (*n* = 4) or (**B**) human peritoneal lesions (NCBI Gene Expression Omnibus GSE179640, *n* = 6) was highly significant, with the hypergeometric test *P* value less than 10^–13^ for both comparisons. (**C**) Similar enrichment (GSEA FDR *P* < 0.05) for increased NFKB, inflammatory response, and hypoxia genes in ES (*n* = 6) and baboon endometriotic lesions (*n* = 7).

To further determine the suitability of ES to model lesions in vitro, the transcriptome results were compared with data from baboon (Supplemental Figure 4) or human endometriotic lesions (NCBI Gene Expression Omnibus GSE179640) (29). Over 10,000 genes expressed in baboon or human endometriotic lesions were captured by ES, resulting in highly significant overlaps (*P* < 10^–13^) for commonly expressed genes (Figure 4, A and B). Moreover, 11,161 genes overlapped between baboon and human endometriotic lesions (Supplemental Figure 4A), in support of the baboon model. We identified 2,518 genes only in baboon lesions and 1,083 in the spheroids. The 2,518 genes unique to baboon lesions were characterized based on biological process Gene Ontology (GO) terms and were enriched for immune system processes (Table 1), pointing to the absence of immune cells in vitro. Indeed, leukocyte migration was a top GO term enriched for genes increased in baboon lesions compared with matched endometrium (Supplemental Figure 4D). The MSigDB hallmark gene set (Broad Institute) summarizes 50 specific well-defined biological states or processes and was used to compare the spheroids with baboon tissues by GSEA. Similar altered gene sets were identified in ES and baboon endometriotic lesions (Figure 4B). Specifically, TNF signaling via NFKB, inflammatory responses, and hypoxia associated genes were increased in ES and lesions, while interferon alpha response was decreased in ES and increased in lesions. Immune-related gene sets allograft rejection and IL6 JAK/STAT3 signaling were increased in baboon lesions but absent from the spheroids, mirroring the enrichment of immune process genes unique to lesions (Figure 4C).

### ES invade a 3D model of the peritoneum.

**Figure 5:**
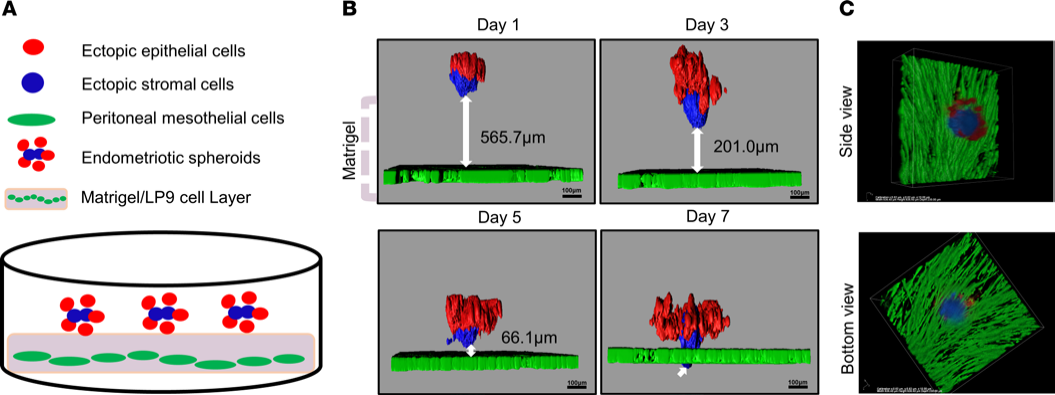
ES invade a 3D model of the peritoneum. (**A**) Schematic of the spheroid invasion model. (**B**) Time course of spheroid invasion quantified with Imaris image analysis software and relative to invasion into the mesothelial cell layer. Ectopic stromal cells were the leading edge of invasion through the LP9 cells. Note the penetration of spheroids through the extracellular matrix and mesothelial cell layer in a time-dependent manner. (**C**) Representative side view and bottom view of an ES invading the LP9 cells with stromal cells at the leading edge on day 8.

Cell invasion is one of the key processes involved in endometriotic lesion initiation and development. To simulate cell invasion that occurs in vivo, we developed a 3D ES invasion model. As shown in Figure 5A, human peritoneal mesothelial cells, LP9 cell line, expressing GFP were grown in Matrigel to mimic the structure of the peritoneum. ES containing iEc-ESC and 12Z were seeded on the top of the LP9/Matrigel layer. The invasion of spheroids was tracked using fluorescence confocal microscopy every other day for 7 days. The *Z*-stack images were reconstructed to produce 3D models with Imaris image analysis software by Bitplane. As shown in Supplemental Video 2 and Figure 5, B and C, the spheroids invaded the Matrigel and continued penetrating the LP9 cell layer. Interestingly, stromal cells were always noted at the leading edge of invasion and as the first cell type to penetrate the LP9 cell layer. The distance between the leading edge of spheroids and the mesothelial cell layer was measured at each time point.

### The effect of steroid hormones on the invasion of ES.

**Figure 6:**
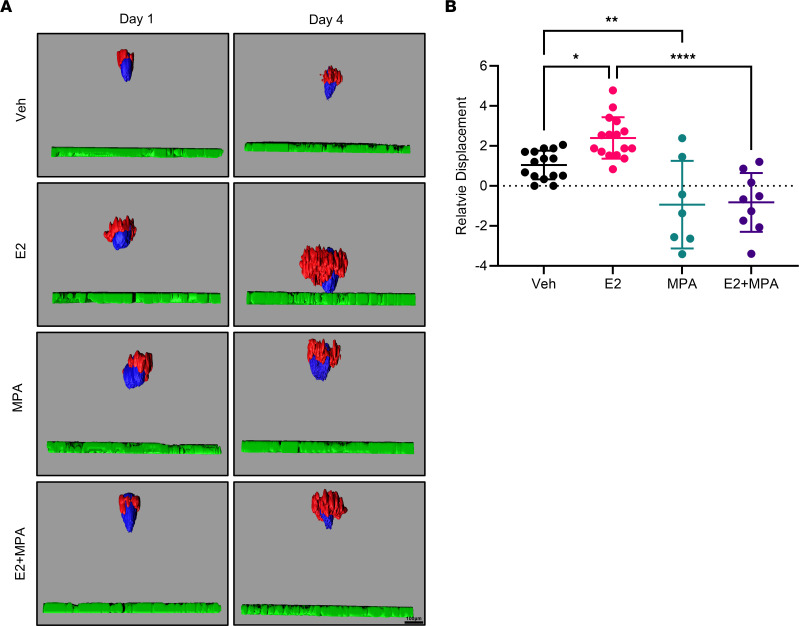
ES invasion is hormone responsive. (**A**) Day 4 ES were seeded on an LP9/Matrigel layer and treated with vehicle (ethanol, *n* = 15), estradiol (E2, 20 nM, *n* = 16), medroxyprogesterone 17-acetate (MPA, 1 μM, *n* = 7) or E2+MPA (*n* = 9) for 4 days. The ES were imaged on days 1 and 4 using fluorescence confocal microscopy, and invasion, relative to into the LP9 cell layer, was quantified. (**B**) Invasion was increased by 2.3-fold in the presence of E2 while decreased by 1.9-fold in the presence of MPA (comparison of MPA with Veh). Statistical analysis for spheroid invasion was performed using the 1-way ANOVA (*P* < 0.05) with post hoc Tukey’s test. Data are shown as mean ± SD. *P < 0.05, **P < 0.01, ****P < 0.0001.

Endometriosis is an estrogen-dependent disease and estrogen can promote the migration and invasion of endometrial stromal cells and endometriotic epithelial cells in 2-dimensional culture (30, 31). Medroxyprogesterone acetate (MPA), a progestin, can block estrogenic effects. The effect of estrogen on ES is unknown, but we hypothesized that estradiol would stimulate invasion in our model. Invasion of day 4 ES through the Matrigel/LP9 cell layer was monitored in the presence of 20 nM estradiol (E2), 1 μM MPA, E2 combined with MPA, or vehicle for 4 days. Spheroids were imaged on day 1 and day 4 by fluorescence confocal microscopy. The invasion rate of ES was increased at 2.3-fold in the presence of E2 compared with vehicle yet was decreased at 1.9-fold in the presence of MPA (Figure 6). In addition, the effect of estradiol on spheroid invasion was inhibited in the presence of MPA.

### Pro-inflammatory macrophages increase ES invasion.

**Figure 7:**
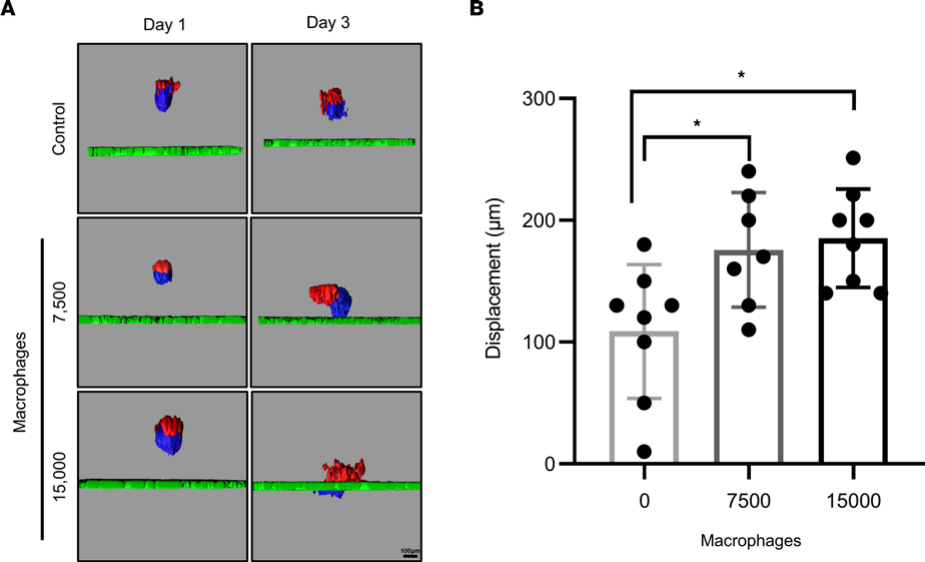
Pro-inflammatory macrophages increase ES invasion. (**A**) Day 4 ES were seeded on an LP9/Matrigel layer and M1 macrophages (7,500 or 15,000) were added to the plates after 3 hours. Control *n* = 8; M1 7,500 *n* = 7; M1 15,000, *n* = 8. The ES were imaged on days 1 and 3 using fluorescence confocal microscopy, and (**B**) invasion, relative to the LP9 cell layer, was quantified. Statistical analysis for spheroid invasion was performed using the 1-way ANOVA (*P* < 0.05) with post hoc Tukey’s test. Data are shown as mean ± SD. *P < 0.05.

Immune cell dysfunction and local inflammation in the endometriotic environment have been considered to play a pivotal role in the pathogenesis of endometriosis (32). The recruitment of macrophages within the endometriotic lesion has been demonstrated to facilitate the development and maintenance of endometriosis (33–35). In support of a role for macrophages in the pathophysiology of endometriosis, immunoreactive CD68, a pan-macrophage marker, was detected in a baboon endometriotic lesion (Supplemental Figure 5). Pro-inflammatory, or M1, macrophages have the capacity to secrete inflammatory cytokines, including IL-6. These inflammatory cytokines may further promote endometriotic cell growth and invasion (36). To address the effect of activated pro-inflammatory macrophages on ES invasion, M1 were differentiated from naive (M0) macrophages derived from the THP-1 cell line by exposure to IFN-γ and lipopolysaccharide (LPS) for 24 hours. The resulting pro-inflammatory macrophages were acclimatized for 24–36 hours in the spheroid culture medium. ES were plated on the top of the Matrigel/LP9 layer, and pro-inflammatory macrophages were added to the wells 3 hours later. The ES were imaged on days 1 and 3 using fluorescence laser-scanning confocal microscopy. Coculture with either 7,500 or 15,000 macrophages increased ES invasion by 1.6- or 1.7-fold, respectively, and there was no significant difference between the macrophage treatment groups (Figure 7). Thus, the addition of pro-inflammatory macrophages promoted the ES invasion, in support of the hypothesis that macrophages facilitate the establishment of endometriotic lesions.

## Discussion

A 3D spheroid model consisting of both epithelial and stromal cells holds promise as an excellent model for dissecting mechanisms associated with early endometriotic lesion development. Most cases of endometriosis are hypothesized to be the result of retrograde menstruation of endometrial fragments containing both epithelium and stroma (37, 38). Therefore, inclusion of both cell types more closely resembles the pathophysiology of endometriosis and advances the potential for in vitro discovery. Ultra-low-attachment plates and hanging-drop culture have been used to generate spheroids with epithelial 12Z cells, immortalized stromal cells, primary ectopic stromal cells, or a combination of epithelial and stromal cells to investigate endometriosis (39, 40). Interestingly, both of these culture models form self-organizing spheroid structures, but separate epithelial and stromal cell compartments are evident only in ultra-low-attachment conditions (39), indicating that ultra-low-attachment culture, which mirrors the compartmentalization seen in vivo, might be more suitable for developing spheroids with multiple cell types.

In this study, we developed ES with endometriotic epithelial and stromal cell lines using a micro-molded agarose plate culture system. Spheroids self-organized and resulted in a stromal cell core surrounded by epithelial cells, a morphology that is similar to that of endometrial spheroids derived from primary endometrial cells (16). Interestingly, the origin of the stromal cells, normal or endometriotic, altered the spheroid structure, with stratified epithelial cells forming only with ectopic stromal cells. The observed epithelial stratification is consistent with reports of endometriotic epithelial spheroids grown in Matrigel (18) and suggests that the ectopic stromal cells promoted epithelial cell proliferation and loss of apical-basal polarity. Indeed, immunohistochemistry results from ES for the proliferation marker Ki67 showed positive staining in the outer layers where the 12Z cells were located (Supplemental Figure 1G). Transcriptomic analyses revealed that pathways dysregulated in endometriotic lesions were present in ES, supporting the idea that ES reproduce a similar transcriptomic profile to that associated with endometriosis. Of note, normal endometrial epithelial cell lines were not included in this study and are not currently readily available, although a simian virus 40–transformed endometrial epithelial cell line, hEM3, was recently reported (41). Therefore, some of the gene signatures obtained from the classic comparison of endometriotic lesion versus normal endometrium may be absent from our study because of the limitation that both spheroid types included diseased epithelium. This comparison, however, highlighted the effect of stromal cell origin on epithelial phenotype and poses important questions regarding the contribution of each cell compartment to the pathophysiology of lesions. Overall, our results support the idea that ES recapitulate multiple properties of endometriotic lesions with respect to cell type compartmentalization and gene expression.

Attachment and invasion are important steps in the development of endometriotic lesions (7). Most current methods to study cell invasion are based on single cell types in 2-dimensional culture or Transwell assays. We developed a 3D spheroid invasion model based on peritoneal endometriotic lesions to more accurately simulate the cell invasion process that occurs in vivo. To our knowledge, this is the first 3D invasion model that includes the 3 main cell compartments, epithelium, stroma, and peritoneal mesothelium. ES migrated through a layer of Matrigel, contacted, and penetrated through a layer of mesothelial cells to mimic peritoneal invasion. Interestingly, our results identified stromal cells at the leading edge of invasion despite their location at the center of the spheroid. This observation agrees with previous studies investigating adhesion and invasion of endometrium to peritoneum using both immunocompromised mice and ex vivo tissue explant culture models (42, 43). Endometrial stromal cells from women with endometriosis undergo changes in adhesive properties and mesenchymal characteristics in response to peritoneal mesothelial cells that might be part of the underlying mechanisms of lesion initiation (44). Our data indicate that ES have the capacity to invade a monolayer of mesothelial cells in a manner hypothesized to occur in physiological conditions. The utility of this model was further supported by increased invasive capacity in the presence of E2 or activated proinflammatory macrophages while the effect on invasion was inhibited in the presence of MPA. Collectively, this model expands the possibilities to study mechanisms of endometriotic lesion establishment in vitro.

Stromal cell origin significantly altered the transcriptome of ES, with over 4,500 DEGs. The genes expressed by ES largely mirrored those from spontaneous endometriotic lesions in the baboon, and GSEA validated coordinated increases in TNF signaling via NF-κB, inflammatory response, and hypoxia-associated gene sets. Genes associated with processes in endometriotic lesions, increased cellular stress, inflammation, proliferation, and senescence, were enriched in ES. A decrease in stem cell–related genes was unexpected. The leading-edge genes contributing to the finding were enriched in extracellular matrix GO terms for molecular function, biological process, and cellular component categories including COL1A2 (fold-change = –2.87, FDR *P* = 1.87 × 10^–7^) and COL5A2 (fold-change = –1.55, FDR *P* = 6.02 × 10^–11^). The original report for this gene set described increased transcripts related to cartilage in adipose stem cells (45). Thus, expression of extracellular matrix genes in ES was the major contribution to the overlap with the stem cell genes. These observations support the utility of ES containing both endometriotic epithelial and stromal cells.

Analysis of spheroid transcriptome alterations at the gene level overlapped with several pathways known to be altered in endometriotic lesions, for example, retinoic acid (RA) signaling, both the uptake of retinoid (46) and the production of RA (47). Several enzymes in the RA pathway were altered in ES, including decreased expression of *STRA6* and *CRABP2* (48) and increased *CYP26B1*. *TERT* expression was increased in ectopic spheroids and among the most highly upregulated genes. However, *TERT* was not altered in a comparison of stroma-only spheroids (Supplemental Figure 2), suggesting that the upregulation in ES was a result of epithelial-stromal interactions. Increased expression of *TERT* has been reported in endometriosis from eutopic secretory endometrium (49–53) and endometriotic lesions (54). Therefore, the relevance of increased expression in ES was supported by increased *TERT* in human tissues. Since these results highlighted the importance of epithelial-stromal interactions, future studies will explore signaling between the compartments with spatial transcriptomics of endometriotic lesions and RNA sequencing from flow-sorted spheroid cells. In further support of the model, endometriotic lesions are progestin resistant (55), and concordantly, progesterone receptor expression was downregulated in ES compared with spheroids made with a normal stromal cell line. The expression of *LIF* was increased in ES, possibly due to local estrogenic activity (56) or inflammatory signals (57, 58). These results demonstrate significant alignment of the ES transcriptome with spontaneous disease, including specific dysregulated pathways in endometriotic lesions.

The immune system plays an important role in the establishment and progression of endometriosis (36, 59, 60). Tissue-resident immune cells are refluxed with menstrual tissue, and additional cells are recruited into the peritoneum, where they interact with endometriotic lesions (61). The pro-inflammatory cytokine *IL6* is common to all 3 coordinately increased hallmark gene sets from ES and spontaneous endometriotic lesions from baboons (data not shown). IL-6 expression was increased in the spontaneous baboon lesions and ES and is associated with chronic inflammation, endometriosis, and the presence of macrophages (33, 61–63). IL-6 activates the expression of *CCL2* (also referred to as monocyte chemoattractant protein-1 or *MCP1*) in endothelial cells to recruit monocytes (64), and IL-6 is further produced by the recruited macrophages and dendritic cells. Indeed, macrophages affect lesion establishment and growth in a mouse model of endometriosis (33, 60, 61), and peritoneal macrophages from women with endometriosis produce more IL-6 (65). Genes expressed in spontaneous lesions, but absent from ES, were enriched in immune-related pathways, indicating a role for immune cells in the pathophysiology of endometriotic lesions that was absent in the model. Importantly, the addition of proinflammatory macrophages to the model of peritoneal invasion increased ES invasive capacity. THP-1 is a human leukemia monocytic cell line derived from a male donor (66), so continued studies should use monocytes from female donors to avoid any potential impact from the donor’s sex. The mechanism by which macrophages altered ES invasion is undetermined, but IL-6 may be a key cytokine for the crosstalk between endometriotic lesions and infiltrating macrophages. Our spheroid model is an ideal system to uncover the crosstalk between lesions and immune cells and is a subject for future studies.

We have described a scaffold-free, self-organized ES, composed of both endometriotic epithelial and stromal cells, combined with a model of attachment and invasion of the peritoneum. Our findings support the idea that 3D culture systems allow for the study of complex cell interactions and pathologically relevant processes like invasion and are a promising preclinical tool to evaluate therapeutic treatments for endometriosis. The spheroids recapitulated many of the transcriptomic features of spontaneous peritoneal endometriotic lesions from the baboon, and the model allowed for real-time imaging and quantification of spheroid invasion. The complexity of this model could be increased by altering conditions with exogenous compounds or other cell types. Future studies utilizing this system can capitalize on this flexibility with combinations of additional cell types, cell type genomic editing, and pharmacological compounds. At present our in vitro model does not reflect the full complexities of macrophage populations observed in vivo (33, 60, 61), and future experiments should investigate the interaction of peripheral monocytes or naive macrophages on ES to determine the effect on all cell types, including the resultant macrophage phenotypes. Furthermore, ES could be integrated with microfluidics cultures containing endometrium (67, 68) to study endometriosis-associated endometrial receptivity failure and decidualization defects.

In conclusion, our study suggests that ES recapitulate many characteristics of endometriotic lesions and supports the concept that spheroids are an improved model for dissecting the mechanisms that contribute to endometriotic lesion establishment.

## Methods

### Cell lines.

Immortalized human endometriotic epithelial cells (12Z cells; a gift from Anna Starzinski-Powitz at Goethe University, Frankfurt, Germany) (27) were cultured using Dulbecco’s modified Eagle’s medium (DMEM)/F-12 (Gibco) supplemented with 10% charcoal dextran–treated fetal bovine serum (CDS-FBS; Gibco), 1× penicillin/streptomycin (Pen/Strep) (Gibco), and 1× sodium pyruvate (Gibco) (27, 69). The human peritoneal mesothelial cell line LP9 was provided by James G. Rheinwald (Harvard Medical School, Boston, Massachusetts, USA). LP9 cells were maintained in a 1:1 ratio of M199 and Ham’s F12 media (Gibco), supplemented with 15% FBS, 1% Pen/Strep, 1% HEPES (Gibco), 1% GlutaMAX (Gibco), 10 ng/mL epidermal growth factor (MilliporeSigma), and 400 ng/mL hydrocortisone (MilliporeSigma). The immortalized human endometriotic stromal cell line (iEc-ESC) was established in our laboratory (28). iEc-ESCs were cultured with phenol red–free DMEM/F-12 medium supplemented with 10% CDS-FBS, 100 U/mL of Pen/Strep, and 1 mM sodium pyruvate. An immortalized human uterine fibroblast cell line (iHUF) was established from the decidua parietalis from term placenta in our laboratory. iHUF cells were cultured in phenol red–free RPMI 1640 (Thermo Fisher Scientific) supplemented with 10% CDS-FBS, 100 U/mL Pen/Strep, and 1 mM sodium pyruvate. THP-1 cells (provided by Margaret Petroff at Michigan State University, East Lansing, Michigan, USA) were grown in a T75 flask using RPMI 1640 with 10% FBS in the presence of antibiotic-antimycotic (Gibco) in the suspension culture. These cells were subcultured either in 6-well plates or a T25 flask and treated with 100 nM (1:5,000 μL of media) PMA (Cayman Chemicals) to yield 60%–70% adherent cells at the end of 24 hours. The PMA exposure was repeated, and any floating, nonconfluent cells were removed. The cells obtained following PMA treatment were designated as M0 macrophages and kept in a predifferentiated (M0) state for 2–4 days before exposing them to M1/M2 differentiation. These M0 adherent macrophages were activated with 20 ng/mL IFN-γ (R&D Systems) and 10 pg/mL of LPS (MilliporeSigma) for 24 hours to differentiate into M1 pro-inflammatory macrophages utilized to study effects of pro-inflammatory M1 macrophages on the ES culture system. All cell lines were maintained at 37°C in an atmosphere of 5% CO_2_ in air.

RFP lentiviral vector (Ploc-RFP, Horizon Discovery, OHS5832) was used to generate the fluorescently tagged 12Z-RFP cell line. EGFP lentiviral vector (pHX-EGFP, provided by Meenhard Herlyn at The Wistar Institute, Philadelphia, Pennsylvania, USA) was used to generate LP9-GFP stable cell lines. iEC-ESCs and iHUF cells were tagged with Azurite blue using pLV-Azurite (Addgene plasmid 36086). Lentiviral transductions were performed according to manufacturers’ protocols, and successfully tagged cells were further sorted on a Beckman Coulter MoFlow Astrios.

### Generation of ES.

Spheroids were generated in nonadherent, 96-well agarose micro-molds (MilliporeSigma, Z764043) based on the methods described for endometrial organoids (16). 12Z and stromal cells were trypsinized in the flask, and cell suspensions were centrifuged at 200*g* for 5 minutes. Both epithelial and stromal cell pellets were then suspended in complete MammoCult human medium (STEMCELL Technologies, Inc., 05620) supplemented with hydrocortisone and heparin as per the manufacturer’s instructions and 1% Pen/Strep to similar densities. Epithelial and stromal cells were mixed at a 1:50 ratio by volume, and 50 μL of epithelial-stromal cell suspension (1.2 × 10^6^ cells/mL) was added into the center of 1.5% (*w/v*) agarose 96-well (400 μm diameter) micro-molds in 24-well tissue culture plates. Media (400 μL) were added to the culture plate, outside the micro-mold, and changed every 2–3 days. Spheroids with normal uterine fibroblasts (12Z+iHUF) were used as a control since a normal endometrial epithelial cell line is not readily available.

### Immunofluorescence staining.

Agarose micro-molds containing spheroids were removed from the tissue culture plate, and the medium inside was removed gently by pipetting under a dissecting microscope. Spheroids were sealed within the agarose dish with lukewarm 1.5% (*w/v*) agarose in 1× PBS. The micro-molds containing spheroids were then fixed in 4% paraformaldehyde in 1× PBS at 4°C overnight, rinsed briefly in 1× PBS, stored in 70% EtOH, and processed for standard paraffin embedding and sectioning at 6 μm. Spheroid sections were dewaxed, then rehydrated with a graded alcohol series, followed by heat-mediated antigen retrieval in citrate buffer (Vector Laboratories, H3300) and then hydrogen peroxide treatment. Sections were blocked for 1 hour in 10% normal horse serum (Vector Laboratories, S-2000) in PBS and incubated overnight at 4°C with primary antibodies. Spheroid sections were incubated in species-specific fluorochrome-conjugated secondary antibodies following an overnight incubation with the primary antibodies. Slides were then mounted with Antifade Mounting Medium with DAPI (Vector Laboratories, H-1200). Images were captured with a Nikon Eclipse Ti inverted microscope using software NIS-Elements (Nikon). The primary antibodies used were vimentin polyclonal antibody (5741S) from Cell Signaling Technology, Inc. and cytokeratin, pan (mixture), monoclonal antibody (C2562) from MilliporeSigma. The secondary antibodies were DyLight 594 goat anti–rabbit IgG antibody (DI-1594) from Vector Laboratories and Alexa Fluor 488 AffiniPure donkey anti–mouse IgG (715-545-150) from Jackson ImmunoResearch Laboratories Inc.

### Animal studies.

In the cycle before planned induction of endometriosis (70), baboons (*Papio anubis*; Charles River Laboratories International, Inc.) were examined for spontaneously occurring disease. Four animals were diagnosed with spontaneous disease and were collected on day 10 postovulation (midsecretory phase). At necropsy, endometrium and endometriotic lesions were collected, and samples were snap-frozen in liquid nitrogen for RNA extraction.

### RNA-sequencing and data analysis.

The Arcturus PicoPure RNA Isolation Kit (Thermo Fisher Scientific, 12204-01), including an on-column DNA digestion using the RNase-Free DNase Set (QIAGEN, 79254), was used to purify RNA from whole day 7 spheroids. Total RNA was isolated from snap-frozen tissues homogenized in TRIzol reagent (Thermo Fisher Scientific, 15596018) following the manufacturer’s instructions. RNA was stored at –80°C in nuclease-free water, and concentration was determined with a NanoDrop 1000 instrument (Thermo Fisher Scientific). Samples were sent to a sequencing facility (Novogene Corporation Inc.) for RNA integrity analysis, library preparation, and sequencing. Libraries were prepared with an NEBNext Ultra II RNA Library Prep Kit (New England Biolabs) and sequenced (paired end 150 bp) on a NovaSeq 6000 instrument (Illumina Inc.) to an average depth of 39 million fragments per sample. Reads were quality trimmed, adapters were removed using TrimGalore (version 0.6.5) (71), and quality-trimmed reads were assessed with FastQC (version 0.11.7). Trimmed reads were mapped to *Homo sapiens* GRCh38 (hg38) or *Papio anubis* (version 3.0) genome assembly using HISAT2 (version 2.1.0) (72). Reads overlapping Ensembl annotations (version 99) (73) were quantified with featureCounts (version 1.6.2) (74) prior to model-based differential expression analysis using the edgeR-robust method (version 4.0.3) (75) in R. Genes with low counts per million (CPM) were removed using the filterByExpr function from edgeR. Transcriptome-wide read distributions were plotted to ensure a similar distribution across all samples with box plots of log-transformed CPM values. Multidimensional scaling plots, generated with the plotMDS function of edgeR, were used to verify group separation prior to statistical analysis. Principal component analysis of normalized counts was conducted with the prcomp function in R using the top 500 variable genes. DEGs were identified as FDR *P* value less than 0.05.

The hclust package was used for hierarchical clustering (ward.D) of Euclidean distances. Heatmaps were generated using log-transformed TPM in pheatmap (version 1.0.12). Where necessary, baboon Ensembl genes were converted to human Entrez identifiers with the human ortholog table downloaded from BioMart (version 101). RNA-sequencing data from human peritoneal endometriotic lesions were downloaded from NCBI Gene Expression Omnibus project GSE179640 (29), and gene identifiers were converted with the mapIds function from the AnnotationDbi package (version 1.60.0) and annotation from the org.Hs.eg.db package (version 4.2). The phyper function in R was used for hypergeometric tests of overlapping genes expressed at an average TPM > 2 with *m* equal to the number of genes remaining after using the filterByExpr function in edgeR. Gene set enrichment and overrepresentation analyses were completed with clusterProfiler package (version 4.6.0) using MSigDB and KEGG reference databases.

### Spheroid invasion model.

To mimic the structure of the peritoneum, an ice-cold LP9 cell suspension was mixed with ice-cold Matrigel (Corning, 536232) at a ratio of 2:1 (v/v), 30 μL drops of Matrigel-cell suspension were plated into a 96-well plate at a density of 2 × 10^4^ to 2.5 × 10^4^ cells per well, cells were allowed to set at 37°C for 3–4 hours until solidified and were overlaid with 100 μL MammoCult growth medium. The next day, the day 4 ES were harvested from 3D Petri dishes by pipetting and then seeded onto the top of the Matrigel-LP9-GFP layer in 96-well plates in 100 μL MammoCult growth medium. Spheroid invasion into the LP9-GFP-Matrigel layer was imaged every 48 hours using confocal microscopy.

To examine the effect of steroid hormones on the invasion of ES, day 4 ES that were seeded on an LP9/Matrigel layer were treated with either 20 nM E2 (MilliporeSigma, E2257) or 1 μM MPA (MilliporeSigma, M1629) or E2 combined with MPA or vehicle as a control (100% EtOH) for 4 days. Media were changed every other day. To examine the effect of pro-inflammatory macrophages on the invasion of ES, day 4 ES were seeded on an LP9/Matrigel layer, and M1 macrophages (7,500 or 15,000) were added 3 hours later. The number of M1 macrophages used in the coculture was determined by the preliminary experiment. Thus, we decided to increase the number to 7,500–15,000 per well. The ES were imaged on days 1 and 4 using fluorescence confocal microscopy, and invasion relative to the LP9 cell layer was quantified.

### Spheroid imaging.

Spheroids were imaged using a Nikon Eclipse Ti inverted microscope using a Nikon C2+ confocal microscope laser scanner. For each spheroid, *Z*-stacks were generated with a 10 μm increment, and tiled scans were set up to image the entire width and depth of the spheroid with 10× air objective. The position of the each spheroid was recorded as *x*,*y* coordinates by ND Acquisition of NIS-Elements software. For spheroid sections, *Z*-stacks were generated with 0.5 μm increment using 40× oil objective. Images were merged using NIS-Elements C2 version 4.13.

### Quantification of invasion.

Commercial software Imaris ×64 7.4.2 (Oxford Instruments) was used for image analysis. In order to quantify the invasion of the spheroid stromal cells into the peritoneal mesothelial cells, 3D surface models of the LP9-GFP cell layer, and the stromal cells–Azurite blue, were generated. Using the measurement tool in Imaris with orthogonal slicers in *XY*, *YZ*, and *ZX* planes, the linear distance between the spheroid migrating front and the peritoneal cells was calculated. Final distances were subtracted from the initial values to calculate the displacement of spheroids over time. Seven to 10 spheroids per group at each time point were evaluated to account for heterogeneity in spheroid structure as well as movement.

### Statistics.

Unless otherwise stated, statistical analyses were performed using Prism 9.00 (GraphPad Software). All data were expressed as mean ± SD. The Student’s *t* test, 2 tailed, was used for comparisons of 2 groups, and 1-way ANOVA with a least significant difference post hoc test was used for multiple comparisons. A *P* value less than 0.05 was considered significant.

### Study approval.

All experimental procedures were approved by the Institutional Animal Care and Use Committees of the University of Illinois, Chicago, USA (ACC Protocol Number 20-090), and Michigan State University in East Lansing, Michigan, USA.

### Data availability.

Raw FASTQ files were deposited in the NCBI Gene Expression Omnibus as separate spheroid (GSE202661) and baboon experiments (GSE202729).

## Author contributions

YS, GWB, and ATF designed the study. YS, GWB, NRJ, and RA performed experiments. YS, GWB, and RA analyzed data. GWB and ATF acquired funding. ATF was the project administrator. YS and GWB wrote the original draft. YS, GWB, RA, JJK, and ATF reviewed and edited the manuscript. The order of the equally contributing authors was based on the development of the spheroid invasion model.

## Supplementary Material

Supplemental data

Supplemental table 1

Supplemental video 1

Supplemental video 2

## Figures and Tables

**Table 1:**
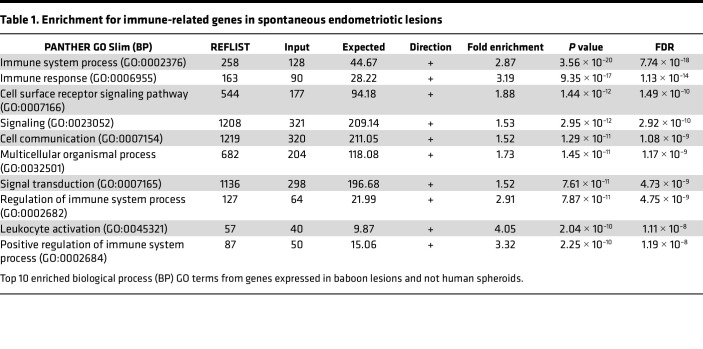
Enrichment for immune-related genes in spontaneous endometriotic lesions

## Version 1 04/27/2023

In-Press Preview

## Version 2 06/08/2023

Electronic publication
