# Supplementary material for: Spheroids as a model for endometriotic lesions
Source: JCI Insight. 2023 Jun 8;8(11):e160815. doi: 10.1172/jci.insight.160815 (PMC10393231; doi:10.1172/jci.insight.160815)
Supplement: Supplemental data [file jciinsight-8-160815-s009.pdf]

Supplemental Data

Spheroids as a Model for Endometriotic Lesions

Yong Song†, Gregory W. Burns†, Niraj R. Joshi, Ripla Arora, J. Julie Kim and Asgerally T. Fazleabas*

Supplemental Figure 1

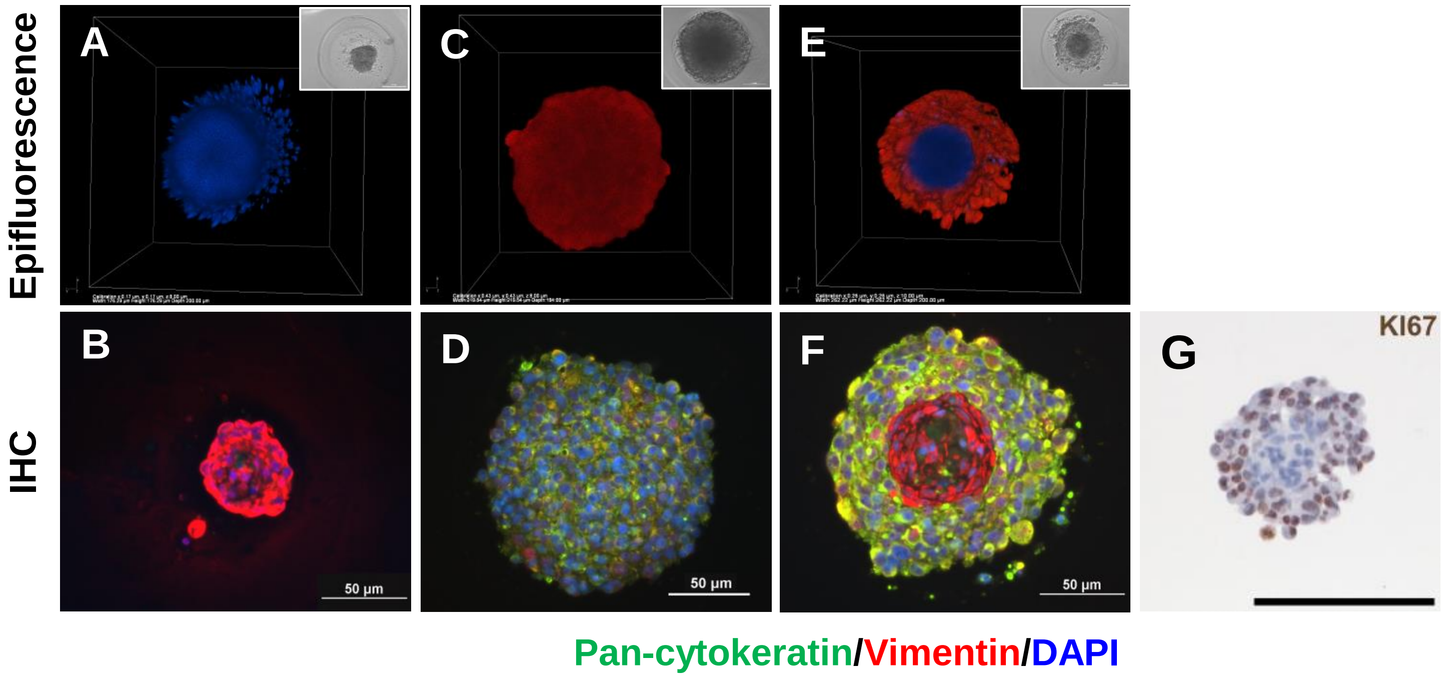

Supplemental Figure 1. Epithelial and stromal cell organization in spheroid culture. spheroids made with (**A-B**) iEc-ESC-Azurite Blue alone, (**C-D**) 12Z-RFP alone, and (**E-F**) a combination of both cell types. (**A**,**C**,**E**) Epifluorescent 3D views of spheroid structure. Inserts are matching phase contrast images. (**B**,**D**,**F**) Immunofluorescent spheroid staining for vimentin (red) and cytokeratin (green). Scale bar = 50 µm (**G**) KI67 positive cells were located on the periphery of endometriotic spheroids, indicating epithelial (12Z) proliferation. Scale bar = 100 µm

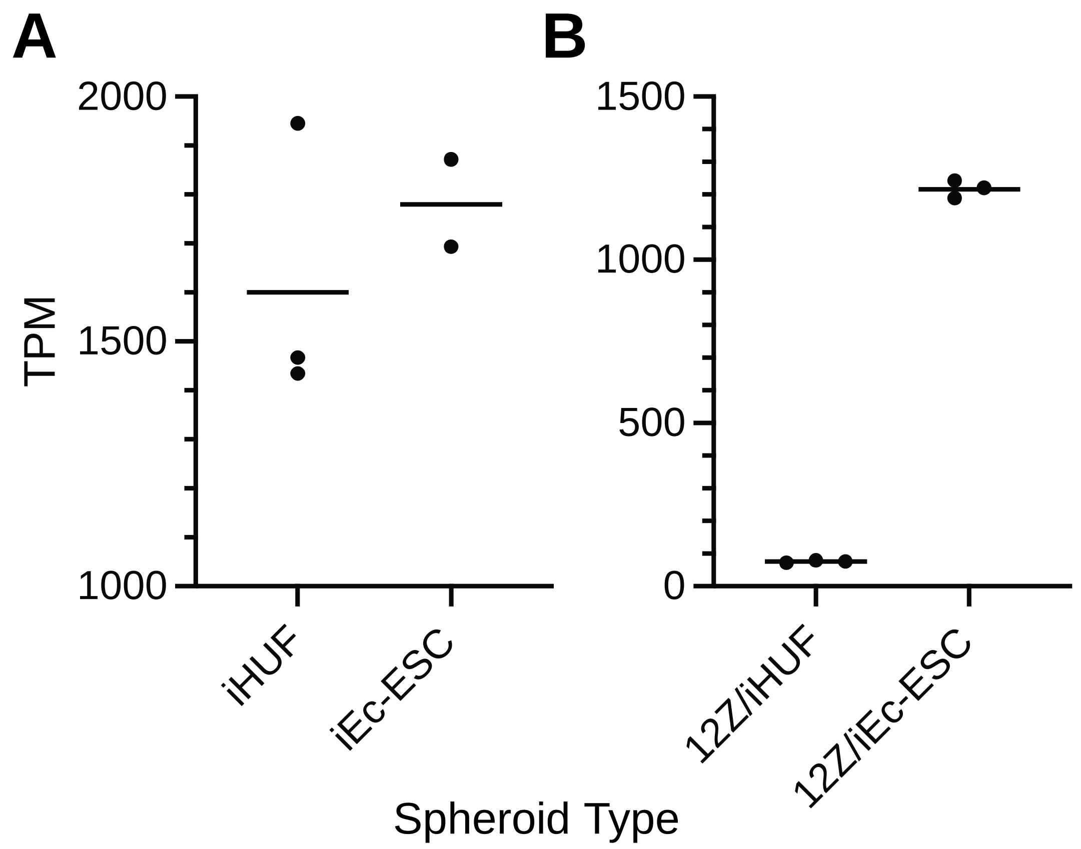

Supplemental Figure 2. Telomerase reverse transcriptase (*TERT*) expression in spheroids. (**A**) Expression was not different between stromal-cell only spheroids (n = 5, edgeR-robust FDR p = 0.91). (**B**) In contrast, *TERT* was one of the most highly increased genes in endometriotic spheroids (12Z/iEc-ESC, FDR p-value = $1.3x10^{-307}$). Horizontal bars are the geometric mean. TPM = transcripts per million

Supplemental Figure 3

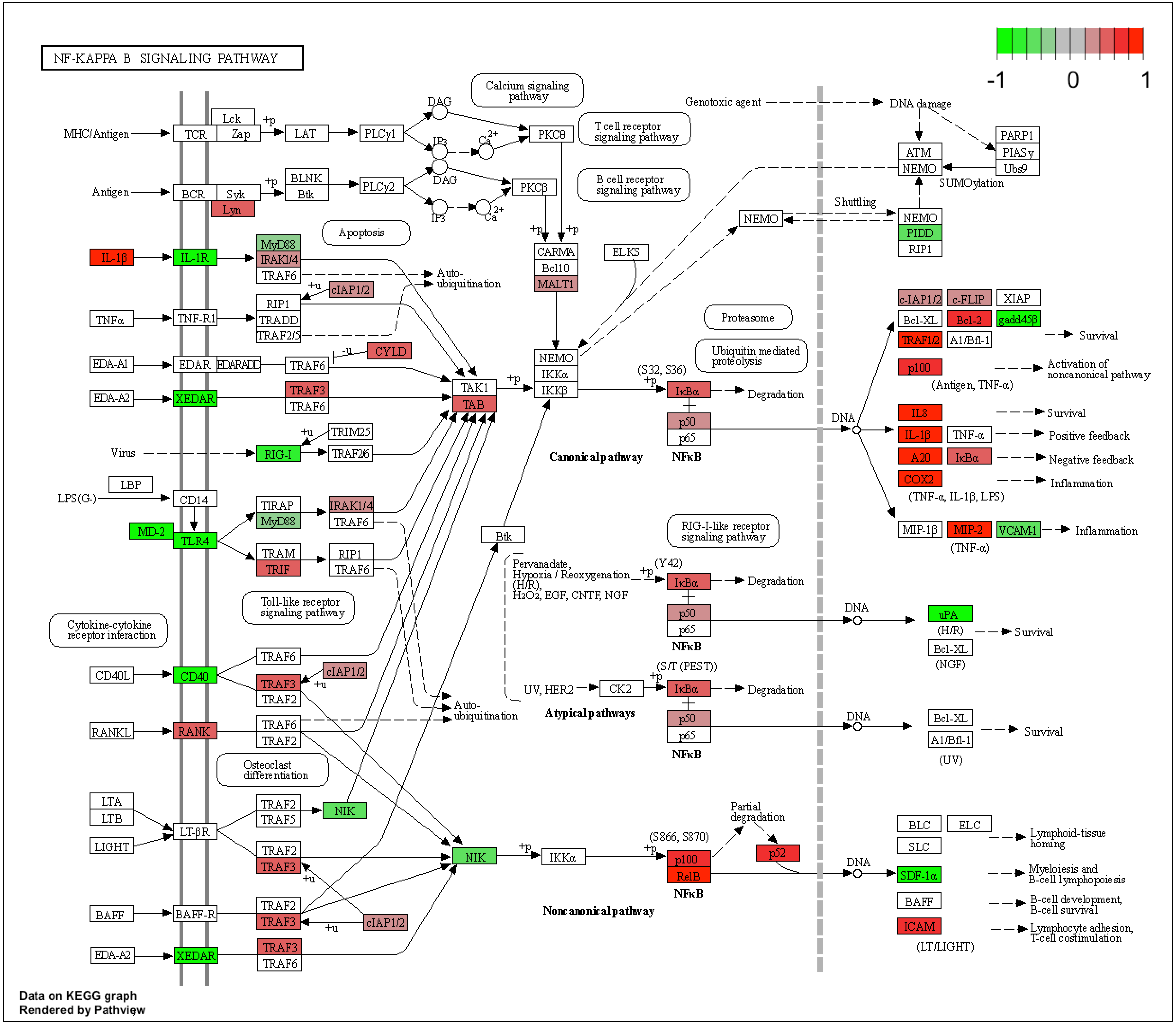

Supplemental Figure 3. NFKB signaling pathway is increased in endometriotic spheroids. Differentially expressed genes from 12Z/iEc-ESC compared to 12Z/iHUF spheroids in the KEGG NFKB signaling pathway are highlighted. Log-transformed fold-change values were used for color coding.

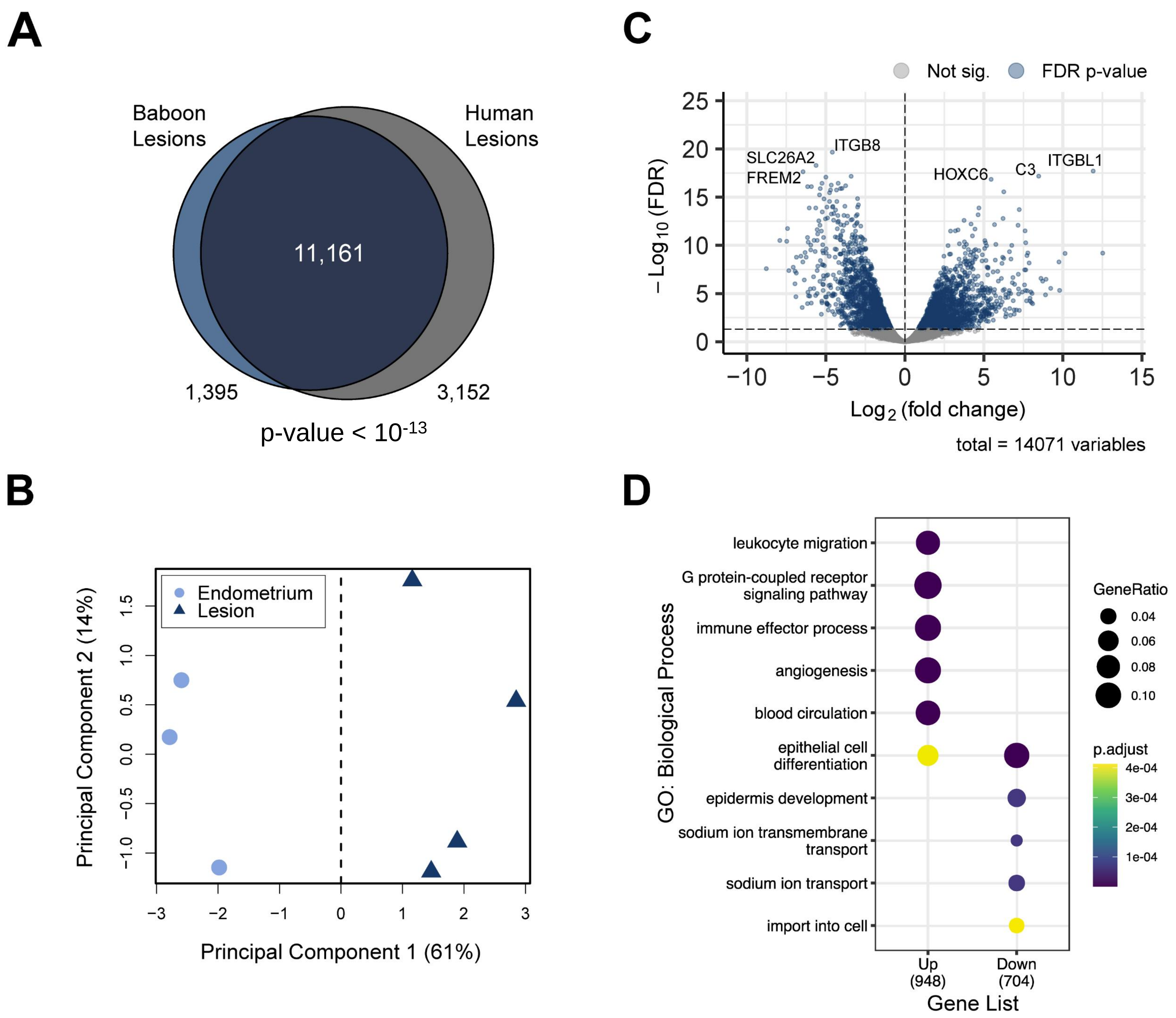

Supplemental Figure 4. Immune-related and angiogenic genes are increased in baboon spontaneous endometriotic lesions. (**A**) Overlap of expressed genes in baboon endometriotic lesions (n = 4) with human peritoneal lesions (GSE179640, n = 6) was highly significant. (**B**) Separation of endometriotic lesions from eutopic endometrium (n = 7) across principal component 1. (**C**) Volcano plot highlighting top differentially expressed genes (edgeR-robust FDR p-value < 0.05). (**D**) The top enriched (hypergeometric FDR p-value < 0.05) biological process terms from up- and down-regulated genes in baboon endometriotic lesions.

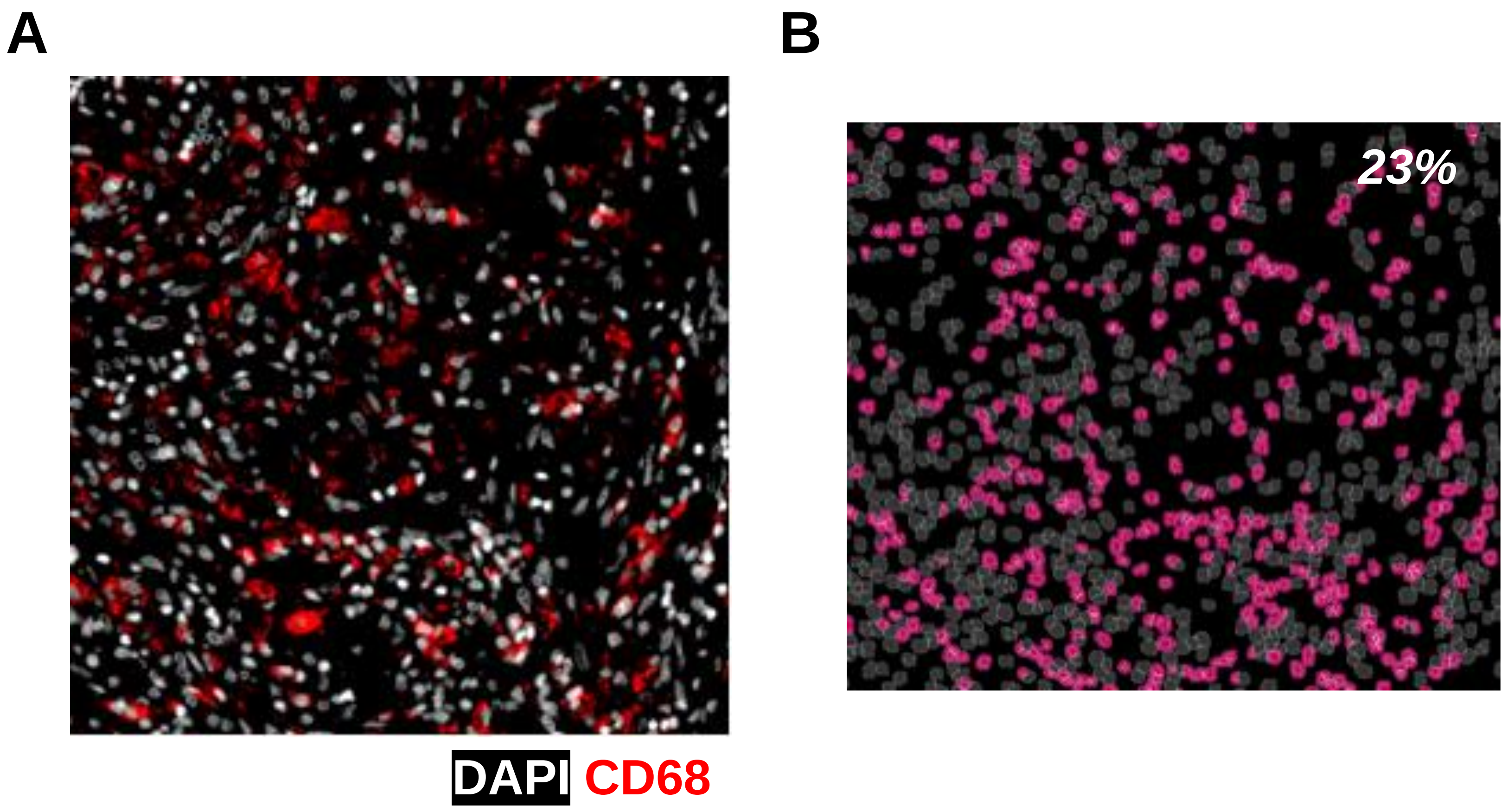

Supplemental Figure 5. Macrophages are present in baboon endometriotic lesions. (**A**) Fluorescent immunohistochemical staining of baboon endometriotic lesions with CD68 (red), a pan-macrophage marker. (**B**) Quantification found 23% of cells were CD68 positive.

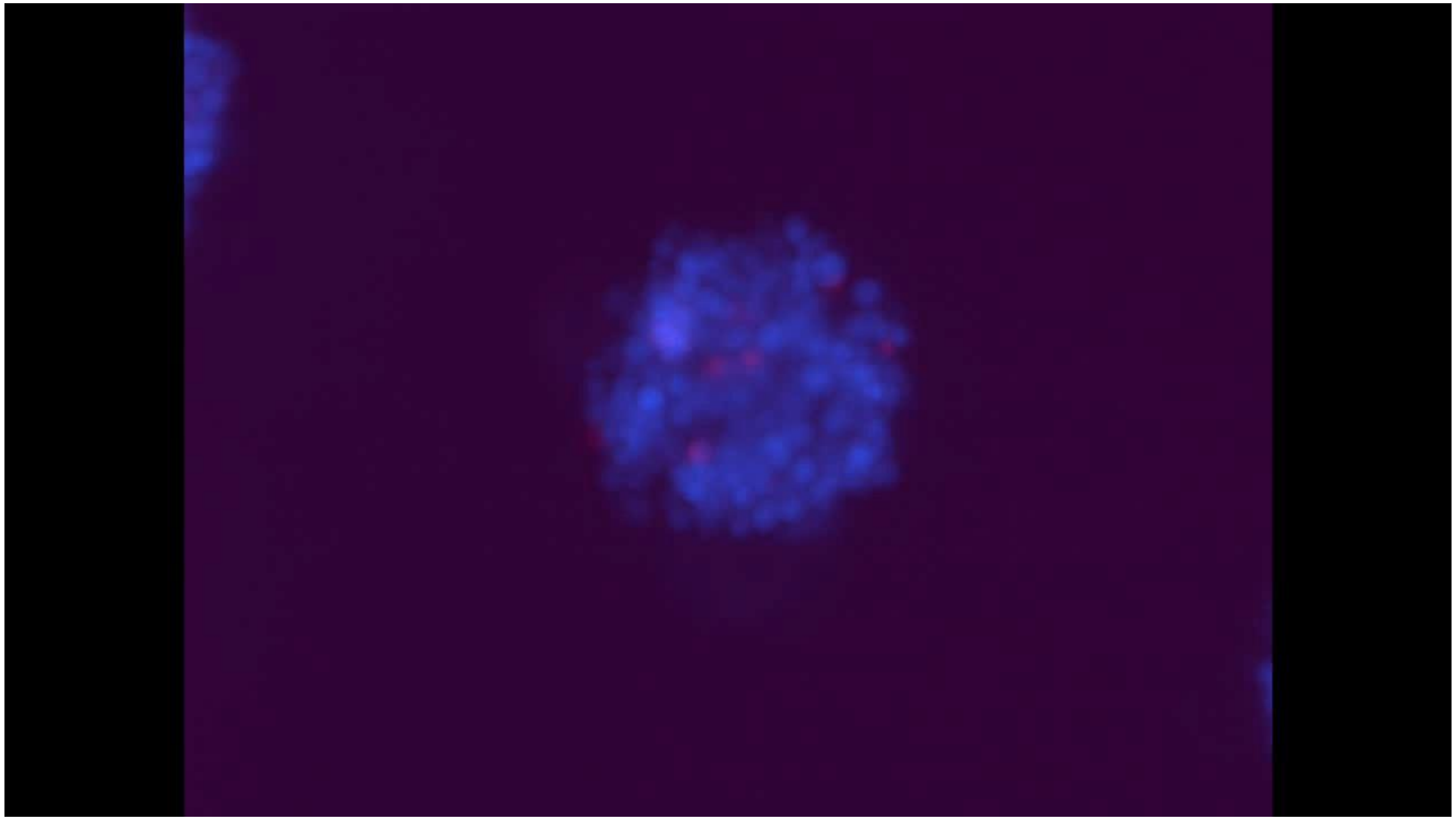

Supplemental Video 1. Endometriotic spheroids self-organize into epithelium and stroma compartments. See related video at https://insight.jci.org/articles/view/160815/sd/1. A representative time-lapse imaging video of ES formation (12Z/iEc-ESC) over the first 3 days of culture. Note the migration of 12Z epithelial cells to the periphery of the stromal cells.

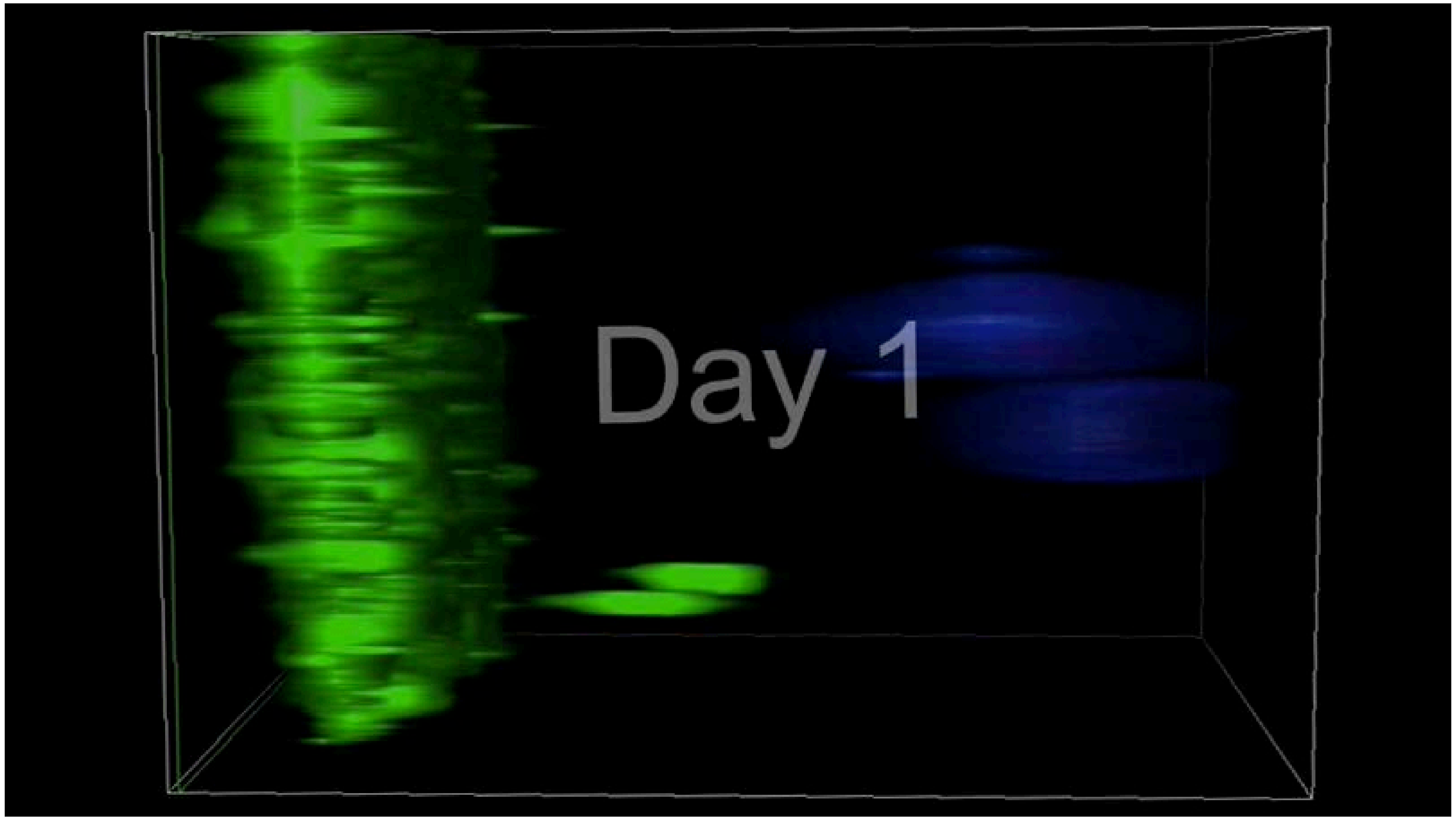

Supplemental Video 2. Confocal live-imaging of ES invasion through a mesothelial cell layer over 8 days. See related video at https://insight.jci.org/articles/view/160815/sd/2. Endometriotic epithelial cells (12Z) express RFP, endometriotic stromal cells (iEc-ESC) express Azurite Blue, and the mesothelial cells (LP9) express GFP.
